# A TCP‐NTCP estimation module using DVHs and known radiobiological models and parameter sets

**DOI:** 10.1120/jacmp.v5i1.1970

**Published:** 2004-05-25

**Authors:** Brad Warkentin, Pavel Stavrev, Nadia Stavreva, Colin Field, B. Gino Fallone

**Affiliations:** ^1^ Department of Medical Physics, Cross Cancer Institute University of Alberta 11560 University Ave. Edmonton Alberta T6G IZ2 Canada; ^2^ Departments of Oncology and Physics, Cross Cancer Institute University of Alberta 11560 University Ave. Edmonton Alberta T6G IZ2 Canada

**Keywords:** NTCP, TCP, radiobiological modeling, treatment planning

## Abstract

Radiotherapy treatment plan evaluation relies on an implicit estimation of the tumor control probability (TCP) and normal tissue complication probability (NTCP) arising from a given dose distribution. A potential application of radiobiological modeling to radiotherapy is the ranking of treatment plans via a more explicit determination of TCP and NTCP values. Although the limited predictive capabilities of current radiobiological models prevent their use as a primary evaluative tool, radiobiological modeling predictions may still be a valuable complement to clinical experience. A convenient computational module has been developed for estimating the TCP and the NTCP arising from a dose distribution calculated by a treatment planning system, and characterized by differential (frequency) dose‐volume histograms (DDVHs). The radiobiological models included in the module are sigmoidal dose response and Critical Volume NTCP models, a Poisson TCP model, and a TCP model incorporating radiobiological parameters describing linear‐quadratic cell kill and repopulation. A number of sets of parameter values for the different models have been gathered in databases. The estimated parameters characterize the radiation response of several different normal tissues and tumor types. The system also allows input and storage of parameters by the user, which is particularly useful because of the rapidly increasing number of parameter estimates available in the literature. Potential applications of the system include the following: comparing radiobiological predictions of outcome for different treatment plans or types of treatment; comparing the number of observed outcomes for a cohort of patient DVHs to the predicted number of outcomes based on different models/parameter sets; and testing of the sensitivity of model predictions to uncertainties in the parameter values. The module thus helps to amalgamate and make more accessible current radiobiological modeling knowledge, and may serve as a useful aid in the prospective and retrospective analysis of radiotherapy treatment plans.

PACS number: 87.53.Tf

## I. INTRODUCTION

Radiotherapy treatment plans are assessed by evaluating the 3D dose distributions calculated by a treatment planning system. Typically, the evaluation process includes the following: (1) looking at the dose distribution superimposed on images of the patient anatomy; and (2) examining dose‐volume histograms (DVHs), which are 1D representations of 3D dose information, for each organ or tumor volume of interest. With these methods of assessment, acceptance or rejection of a plan relies on an implicit estimation of the tumor control probability (TCP) and normal tissue complication probability (NTCP) arising from the dose distribution. This estimation is based on clinical experience with respect to appropriate target doses and corresponding dose‐volume constraints. The advent of more sophisticated radiotherapy techniques such as intensity‐modulated radiotherapy has led to more complex and heterogeneous dose distributions, making such implicit evaluations more difficult. For example, different treatment plans may lead to dose distributions having similar gross dose measures (such as mean dose), but characterized by DVHs with very different shapes. To determine the optimal plan in this case, clinicians may need to rely on relatively vague notions of dose‐volume characteristics of different tissues.

Clearly, a natural application of radiobiological modeling to radiotherapy is the ranking of treatment plans via a more explicit calculation of TCP and NTCP values using models that automatically incorporate the available clinical data regarding the dose‐volume characteristics of different tissues. Unfortunately, the predictive capabilities of current radiobiological models in this regard are still limited.^(1)^ ^,^ ^(2)^ Presently there is still insufficient clinical data on the dose‐response characteristics of human tissues and tumors on which to base reliable estimates of model parameters. This precludes the use of model predictions as a primary evaluative tool. However, such predictions are still a valuable complement to clinical experience. Further, as a result of increased archiving of 3D dose distributions and corresponding treatment outcomes, the quality and quantity of clinical data have begun to improve significantly in the last few years. Since this will surely enhance the reliability of model predictions, it is plausible that radiobiological modeling will play an important role in treatment plan evaluation and optimization in the future.^(3)^

We have developed a convenient software tool for estimating the TCP or NTCP arising from differential (frequency) dose‐volume histograms (DDVHs). The program, TCP_NTCP_CALC, was designed to amalgamate relevant current radiobiological modeling knowledge and make it accessible to clinicians, treatment planners, and researchers. It serves the following functions: (1) as an aid in the prospective evaluation of rival treatment plans, by allowing evaluation and comparison of different model predictions; and (2) as an analysis tool in the retrospective study of radiotherapy treatments that may help establish or repudiate the predictive capabilities of different model/parameter sets.

During the development of our software, we became aware of a software package (BIOPLAN) designed with a similar intent published by Sanchez‐Nieto and Nahum.^(4)^ Although the two packages share some similarities, a number of differences also exist. For example, our module includes the Critical Volume (CV) NTCP model^(^ ^5^ ^–^ ^7^ ^)^ and the recent Zaider‐Minerbo/LQ TCP model,^(8)^ which are not available in BIOPLAN. TCP_NTCP_CALC also includes several different parameter databases and provides a convenient method of archiving (and using) newly published parameter databases. Thus, this additional software is a useful complement or alternative. It is freely available by contacting the authors.

## II. METHODS

### A. Radiobiological models

In general, clinical dose‐response data only have sufficient diversity to support the use of relatively simple radiobiological models; use of complex models with many parameters typically results in significant parameter correlation and ambiguity in biological interpretation. Our NTCP and TCP calculation module incorporates a total of four radiobiological models. Included are two NTCP models: a sigmoidal dose response (SDR) model introduced by Lyman^(9)^ and individual‐based and population‐based variants of the CV model; and two TCP models: a two‐parameter Poisson‐based model and a model employing linear‐quadratic cell kill and the formalism developed by Zaider and Minerbo^(8)^ to account for repopulation. The simple SDR and Poisson models have been most frequently applied in the analysis of normal tissue complication and tumor response data, respectively. The CV NTCP and the Zaider‐Minerbo TCP models are slightly more complex, but are founded on more specific biological descriptions. The four models are briefly discussed in the following paragraphs, and the parameters used in each of the models are summarized in Table 1.

**Table 1:** A list of the parameters and their description for each of the models used in the module. FSU=functional subunit; CV=Critical Volume; LQ=linear‐quadratic.

NTCP model	Parameter	Descriptor of	TCP model	Parameter	Descriptor of
	*n*	dose‐volume relationship		$D_{50}$	position of dose‐response
SDR	*m*	slope of dose‐response	Poisson	γ50	slope of dose‐response
	$D_{50}$	position of dose‐response			
	$\mu_{\text{cr}}$	critical relative volume		*N*	no. of tumor clonogens
CV (individual)	*N*	number of FSUs in organ	Zaider‐Minerbo/LQ	α, β	cellular radiosensitivity – LQ parameters
	α	cellular radiosensitivity		λ	repopulation rate
	$N_{0}$	number of cells in FSU		*n*	number of fractions in treatment
	$\mu_{\text{cr}}$	critical volume			
CV	σ	population variation			
(population)	$D_{50}^{FSU}$	position of FSU dose‐response			
	$\gamma_{50}^{FSU}$	slope of FSU dose‐response			

#### A.1 Sigmoidal dose response NTCP model

The SDR model^(9)^ describes the sigmoidal dose‐response curve of normal tissues using the following probit form:

(1) $$\text{NTCP}=\Phi \left(\frac{\text{EUD}-D_{50}}{mD_{50}}\right),$$

where Φ(*x*) is the probit function

(2) $$\begin{array}{l} \Phi (x)=\frac{1}{\sqrt{2\pi }}\int_{-\infty }^{x}\text{exp}\left(\frac{-t^{2}}{2}\right)dt\\ \;=\frac{1}{2}\left[1+erf\left(\frac{x}{\sqrt{2}}\right)\right], \end{array}$$

with $x=(\text{EUD}-D_{50})/mD_{50}$. In Eq. (1), EUD is the equivalent uniform dose, which represents the dose that, if delivered uniformly to the entire organ, would produce the same effect as the given heterogeneous dose distribution, as specified by the DVH. Here, it is assumed that the EUD is equal to a generalized mean dose (GMD), calculated from the dose‐volume pairs $\left\{D_{i},v_{i}\right\}$ of the DDVH using

(3) $$\text{GMD}={\left(\sum_{i}v_{i}D_{i}^{1/n}\right)}^{n},$$

For the SDR model, the above method of DVH reduction, which reduces a full DVH to a single dose (GMD) delivered to the entire volume, is equivalent^(10)^ ^,^ ^(11)^ to the Kutcher‐Burman (KB) reduction method,^(12)^ which reduces a DVH to a reference dose delivered to an effective fractional volume. Other methods of reducing DVHs to a single dose or volume parameter have been proposed. The work of Cozzi et al.^(13)^ suggested that most current DVH reduction schemes are somewhat error‐prone because they can lead to DVH reductions inconsistent with the expected biological effect. The KB method was found to be one of the more robust of the available DVH reduction schemes.

The SDR model has three parameters: *n, m,* and $D_{50}$; *n* determines the dose‐volume dependence of a tissue and thus accounts for differences in tissue architecture; *m* controls the slope of the dose‐response curve (in the case of homogeneous irradiation); and $D_{50}$ represents the dose at which there is a 50% chance of complication, and thus dictates the position of the dose‐response curve. Although largely phenomenological, the SDR model can be interpreted as predicting the NTCP for a normally distributed population of individuals each having threshold‐like dose‐response behavior.

#### A.2 Critical volume NTCP model

The CV model^(5)^ ^,^ ^(6)^ is based on the premises that organs are composed of functional subunits (FSUs) and that organ function is compromised when a certain critical fraction $\left(\mu_{\text{cr}}\right)$ of these FSUs is damaged. For a uniformly irradiated organ with *N* FSUs and a reserve capacity of *L‐1* FSUs (i.e., $\mu_{\text{cr}}=L/N$), the probability of complication can be expressed mathematically as

(4) $$\text{NTCP}=\sum_{M=L}^{N}\frac{N!}{M!\;(N-M)!}p_{FSU}^{M}(D)\;{\left(1-p_{\text{FSU}}(D)\right)}^{N-M},$$

where $p_{\text{FSU}}(D)$ is the probability of damage to an FSU after receiving a dose *D*. Since the number of FSUs is always quite large, the cumulative binomial distribution in Eq. (4) can be approximated by a cumulative normal distribution^(14)^:

(5) $$\text{NTCP}=\Phi \left(\frac{\sqrt{N}(p_{\text{FSU}}(D)-\mu_{\text{cr}})}{\sqrt{p_{\text{FSU}}(D)\;(1-p_{\text{FSU}}(D))}}\right),$$

where the Φ (probit) function is as defined in Eq. (2). For the case of a heterogeneously irradiated organ, the probability of complication becomes

(6) $$\text{NTCP}=\Phi \left(\frac{\sqrt{N}\left(\sum_{i}v_{i}\;p_{\text{FSU}}(D_{i})-\mu_{\text{cr}}\right)}{\sqrt{\sum_{i}v_{i}p_{\text{FSU}}(D_{i})\;(1-p_{\text{FSU}}(D_{i}))}}\right).$$

Eq. (6) assumes that the total damage to the organ can be treated as the sum of damage to independent subvolumes. In Eq. (6), the sum $\sum_{i}v_{i}p_{\text{FSU}}(D_{i})$ can be identified as the mean relative damaged volume, ${\bar{\mu }}_{d}$. For our implementation of this CV model, the probability of damage to an FSU is calculated using

(7) $$P_{\text{FSU}}(D)={\left(1-e^{-\alpha D}\right)}^{N_{0}}.$$

The parameters α and $N_{0}$ describe the cellular radiosensitivity and the number of cells in the FSU, respectively, and it is assumed that the FSU is only irreparably damaged when all cells are killed.

The above CV model is appropriate for the description of the dose‐response of an individual patient; clinical data, however, describe dose‐response averaged over a population of individuals. We have thus incorporated in our module a “population” variant of the CV model,^(7)^ which takes into account interpatient variability in normal tissue dose‐response. This CV model assumes that the NTCP for an individual is steplike,

(8) $${\text{NTCP}}_{\text{ind}}=\{\begin{array}{ll} 1 & {\bar{\mu }}_{d}\geq \mu_{\text{cr}}\\ 0 & {\bar{\mu }}_{d}<\mu_{\text{cr}} \end{array},$$

that is, a complication will occur (and only occur) if the mean relative damaged volume is greater than or equal to the critical relative volume. Using the DDVH to calculate $\sum_{i}v_{i}p_{\text{FSU}}(D_{i})$, we now assume that the damage to an FSU can be described by a probit (Φ) function parametrized using position and slope parameters, $D_{50}^{\text{FSU}}$ and $\gamma_{50}^{\text{FSU}}$:

(9) $${\bar{\mu }}_{d}=\sum_{i}v_{i}\;p_{\text{FSU}}(D_{i})=\sum_{i}v_{i}\;\;\Phi \left(\sqrt{2\pi }\gamma_{50}^{\text{FSU}}\text{ln}\frac{D_{i}}{D_{50}^{\text{FSU}}}\right).$$

The “population‐averaged” CV model is then formulated by further assuming that interpatient variability is limited to the critical relative volume $\left(\text{mean}=\mu_{\text{cr}}\right)$ and that values for this parameter are log‐log normally distributed in the population with a standard deviation of $\sigma (\sigma \approx -\sigma_{\mu_{\sigma }}/(\mu_{\text{cr}}\ln\mu_{\text{cr}}))$. It can be shown that ${\text{NTCP}}_{\text{pop}}$ can then be represented by a probit function:

(10) $${\text{NTCP}}_{\text{pop}}\approx \Phi \left(\frac{-\text{ln}(-\text{ln}\;{\bar{\mu }}_{d})+\text{ln}(-\text{ln}\;\mu_{\text{cr}})}{\sigma }\right),$$

which is the form that we used for calculation purposes.

#### A.3 TCP model based on Poisson statistics

TCP models generally rely on the assumption that tumor control requires the killing of all tumor clonogens. Poisson statistics predict that the probability of this occurring is

(11) $$\text{TCP}=\text{exp}(-N\;p_{s}(D))$$

where *N* is the initial number of clonogens, and $p_{s}(D)$ is the cell survival fraction after a dose *D*. If it is assumed that cell survival can be described by single‐hit mechanics,

(12) $$p_{s}(D)=\text{exp}(-\alpha D),$$

the expression in Eq. (11) can be rewritten in terms of the two parameters describing the dose and normalized slope at the point of 50% probability of control, $D_{50}$ and $\gamma_{50}$:

(13) $$\text{TCP}={\left(\frac{1}{2}\right)}^{\text{exp}[2\gamma_{50}(1-D/D_{50})/\ln\;2]}.$$

Using the assumption of independent subvolumes, for the case of heterogeneous irradiation, the overall probability of tumor control is the product of the probabilities of killing all clonogens in each tumor subvolume described by the DDVH:

(14) $$\text{TCP}=\prod_{i}\text{TCP}(D_{i},v_{i}).$$

Thus, for a given DDVH $\left\{D_{i},v_{i}\right\}$, the TCP can be calculated using the following two‐parameter TCP formula:

(15) $$\text{TCP}={\left(\frac{1}{2}\right)}^{\sum_{i}v_{i}\text{exp}[2\gamma_{50}(1-D_{i}/D_{50})/\text{ln}\;2]}.$$

The above formula originates from an attempt to predict the TCP for an individual patient from a mechanistic perspective. However, because of its relative simplicity, Eq. (13) [or Eq. (15) for the case of a heterogeneous tumor dose] is often conveniently used to fit clinical data describing the tumor response of a population of individuals. In this case, the parameters $D_{50}$ and $\gamma_{50}$ are phenomenological in nature.

### A.4 TCP model incorporating radiobiological data

Since the application of Eq. (15) is mainly phenomenological, we believed it would be useful to include a second TCP model that is parametrized in terms of fundamental cellular radiation response characteristics. Recently, Zaider and Minerbo^(8)^ derived a conceptually robust expression for TCP that incorporates the effect of tumor repopulation. The original Zaider‐Minerbo expression, valid for any temporal protocol of dose delivery, has been adapted for the case of a fractionated delivery with varying time intervals between fractions by Stavreva et al.^(15)^ This adapted expression predicts that the TCP after the delivery of *n* fractions is

(16) $$\text{TCP}={\left[1-\frac{p_{s}(T_{n})e^{\lambda T_{n}}}{\left(1-p_{s}(T_{n})e^{\lambda T_{n}}\sum_{k=1}^{n-1}\frac{1}{p_{s}(T_{k})}[e^{-\lambda T_{k+1}}-e^{-\lambda T_{k}}]\right)}\right]}^{N},$$

where λ is the rate of cellular repopulation, $T_{k}$ is the time between the *kth* fraction and the first fraction, and $p_{s}(T_{k})$ is the cell survival after the *kth* fraction. Here, cell survival was predicted using the familiar linear‐quadratic (LQ) model. Assuming that there is complete repair of sublethal cellular damage between fractions, the LQ prediction of cell survival after the *kth* fraction is

(17) $$p_{s}(T_{k})=\text{exp}\left(-\alpha \left(\frac{k}{n}D\right)-\frac{\beta {\left(\frac{k}{n}D\right)}^{2}}{k}\right),$$

where α and β are cellular radiosensitivity parameters, *D* is the total dose delivered in the *n*‐fraction treatment, and it is assumed that the dose delivered in each fraction is the same. To treat the case of heterogeneous irradiation, Eq. (16) is used in conjunction with Eq. (14), with each $\text{TCP}(D_{i},v_{i})$ in Eq. (14) being calculated by evaluating Eq. (16) after making the substitution $N\to Nv_{i}$ and using $D\to D_{i}$ in Eq. (17).

### B. Parameter databases

One of the main purposes of the TCP_NTCP_CALC program is to provide a convenient means of accessing and archiving current and future radiobiological knowledge as it pertains to treatment plan evaluation. The program contains parameter databases for three of the models described above: the SDR NTCP model, the CV NTCP model (“population” variant only), and the Poisson TCP model. For each of these three models there are two databases: a “default” one, which cannot be altered by the user, and a “user” database, for which the user is allowed to add and delete database entries via a menu‐driven interface. Each database entry includes the following data: model name, organ/endpoint or tumor/grade descriptor, parameter values (and corresponding confidence intervals if desired), and descriptors of the parameter database and the clinical data on which it is based.

Although there are published parameter estimates for the “individual” CV model for a few single organs, a comprehensive database of parameter estimates for a large number of normal tissues is not available. Thus, no databases for this model are included in our module. However, if the user is more familiar with or prefers the “individual” CV model, the user has the option of using this model by specifying his or her own parameters.

There are no estimates of the various parameters used in the Zaider‐Minerbo/LQ TCP model for clinical tumor response data. Again, however, the user may use the Zaider‐Minerbo/LQ model by specifying his or her own parameter values. This may be valuable when investigating the sensitivity of TCP predictions to parameter uncertainties (e.g., in the values of the LQ radiosensitivity parameters, α and β) and the effects of repopulation defined with parameter λ.

#### B.1 SDR databases

For normal tissues, the first and still largest compilation of dose‐response data is that published by Emami et al.^(16)^ in 1991. These data provide estimates of up to six dose‐volume points—doses leading to 5% and 50% complication rates for irradiation of one‐third, two‐thirds, and all of an organ—for many different normal tissue types. Based on these data, estimates of the SDR model parameters for 27 of these normal tissues were provided by Burman et al.^(17)^ This parameter set comprises the “default” SDR database in our module.

Until recently, works estimating normal tissue complications have almost exclusively relied on the Burman/Emami SDR parameter set. Unfortunately, the Emami et al. data are not statistical in nature; as a result, uncertainties in the parameter values are indeterminate, as are the corresponding uncertainties in the calculated NTCPs. The development of 3D treatment planning systems and the resulting potential for archival of 3D dose distributions with treatment outcome have much improved the quality of clinical data sets, making it more amenable to radiobiological analysis. In recent years, a number of works have provided parameter estimates, including statistical uncertainties, for several different normal tissues. Recent SDR model parameter estimates include those for the parotid gland,^(^ ^18^ ^–^ ^20^ ^)^ the heart,^(21)^ the lung,^(^ ^22^ ^–^ ^24^ ^)^ and the liver.^(25)^ ^,^ ^(26)^ For further details (e.g., treatment techniques, parameters) about the clinical data on which each of these parameter estimates is based, the reader is referred to the original papers. These parameter sets have been included in the “user” SDR database so that the user can delete them if desired, for example, if the user prefers a given parameter set for a specific organ.

#### B.2 Critical volume (“population”) databases

The default database for the population‐averaged CV model incorporates the parameters published by Stavrev et al.^(7)^ for 16 types of normal tissue. Since these estimates are again based on the Emami et al. data, no parameter uncertainties are available. Stavrev et al. noted that the CV model was flexible enough to describe the data not only of “traditional” CV organs such as liver and lung, but also of organs such as spinal cord and stomach that are believed to have a more “critical element” architecture. However, they caution that although the CV model has a biological foundation, extracted parameter values should be considered phenomenological, owing perhaps to a large degree of parameter correlation inherent in the model. There are a few works that provide estimates for the CV model for single organs. Parameters (including uncertainties) for this particular variant of the CV model based on liver data published by Jackson et al.^(5)^ were also extracted in Ref. 7 and have been included in the “user” CV database of our program.

#### B.3 Poisson TCP databases

A large collection of tumor dose‐response parameters ($D_{50}$ and $\gamma_{50}$) extracted from single‐ and multi‐institution tumor data sets from a variety of sources for many different tumor sites and grades has been compiled and published by Okunieff et al.^(27)^ Sixty‐two of the Okunieff et al. entries are included in our “default” Poisson database. $D_{50}$ and $\gamma_{50}$ values for non‐small‐cell lung cancer from Willner et al.^(28)^ and for prostate cancer from Cheung et al.^(29)^ and Levegrün et al.^(30)^ are included in the “user” database.

### C. Program architecture

The TCP_NTCP_CALC software was developed in the MATLAB (The Mathworks Inc., Natick, U.S.A.) programming environment and is designed for use on a Windows‐based computer (Pentium 3 or faster recommended) with MATLAB (version 6 or greater) software installed. TCP_NTCP_CALC has a menu‐driven user interface, designed for convenient, straightforward use. The framework of the program is simple: The user inputs a DDVH; based on user selection from parameter databases or from user input, appropriate parameters for the available radiobiological models are retrieved; NTCP or TCP calculations are performed based on these parameter values; a convenient display of the relevant model predictions and the DVH are provided. Further details of the program functionality are provided below.

#### C.1 Input

The program accepts DDVHs in either of two formats:

1. the DVH file output from the HELAX‐TMS (Nucletron, Kanata, ON) commercial treatment planning system or
2. a two‐column text file of $\left\{D_{i},v_{i}\right\}$ values.

DDVHs can be evaluated either on an individual basis, in which case a single DDVH file is specified by the user, or as a group, in which case the user need only specify the directory in which the DDVH files are located. The former option (“single‐mode”) is suited for using the program as an aid in treatment plan evaluation, while the latter option (“group‐mode”) is convenient when retrospectively comparing actual treatment outcomes of a cohort of patients with radiobiological model predictions.

#### C.2 Parameter selection/retrieval

After selecting the input DDVH file(s), a menu prompts the user (1) to identify the file as either a normal tissue or a tumor DDVH file, and (2) to choose between using parameters stored in the parameter databases or specifying their own values for one or more of the models for calculation of the NTCPs or TCPs. For normal tissues, the user can access the parameter databases in one of two ways. The first method is to select an organ type from a list of the normal tissues present in the databases. If the DDVH is in HELAX‐TMS format, the program will attempt to automatically identify the organ and ask for user confirmation. After determining the organ type, the program retrieves all available parameter estimates in the databases for this organ, which may include parameters for different complication endpoints and for one or both of the SDR and CV (“population”) models. The user can also access the normal tissue databases in a second way, by selecting any number of entries from listings of the four normal tissue databases (SDR‐default, SDR‐user, CV‐default, CV‐user). For tumors, user selection from the databases is facilitated by listings of the 62 Okunieff et al. entries entered in the default Poisson TCP database, as well as the entries residing in the user database.

As mentioned above, the user can instead choose to specify his or her own model parameters for any of the models: SDR, CV (“population”), CV (“individual”), Poisson TCP, and Zaider‐Minerbo/LQ. Confidence intervals for each of the parameters and the confidence level (e.g., 68% or 95%) can be also be entered. This option allows users to test the sensitivity of radiobiological model predictions of NTCP or TCP to different parameter sets and/or parameter uncertainties.

#### C.2 Calculation of the NTCP/TCPs

Using the retrieved parameters and the DDVH $\left\{D_{i},v_{i}\right\}$, NTCPs are calculated using Eqs. (1) and (3) for the SDR model, Eqs. (6) and (7) for the individual CV model, and Eqs. (9) and (10) for the population CV model. TCP predictions are based on Eq. (15) for the Poisson model and Eqs. (14), (16), and (17) for the Zaider‐Minerbo/LQ model.

When parameter uncertainties are available, the corresponding uncertainties in the TCP or NTCP are estimated using the following Monte Carlo method. A large number (500) of sets of parameter values are generated by randomly sampling a probability distribution of values for each parameter of the model. A distribution of NTCP (or TCP) values is then generated by evaluating the NTCP (or TCP) for each of the sampled parameter sets. The standard deviation of this NTCP (or TCP) distribution is calculated to furnish a measure of the uncertainty in the predicted NTCP (or TCP) value. As described by Schilstra et al.,^(31)^ the probability distribution for each parameter should be related to the value of the likelihood function in a maximum likelihood fitting analysis. The shape of the likelihood contour is, however, unavailable in this case, since only the confidence interval and confidence level are specified. We thus assumed that parameter values were normally distributed, equivalent to assuming that the likelihood function has a normal shape with respect to the model parameters. The confidence interval and level are used to determine the width of the distribution. Since the above assumption is not always valid, the provided NTCP (or TCP) uncertainties should be treated as approximate indicators of the degree of confidence one should have in the different model predictions.

#### C.4 Calculation of the probability distribution of the expected number of complications

When the user chooses to input a group of DDVH files for analysis (“group‐mode” of the program), in addition to calculating the mean NTCP (or TCP) for this cohort, the program will also calculate the probability of observing any number of complications (or controls). This provides an additional and more indepth characterization of the radiobiological modeling predictions than use of the mean NTCP (or TCP) alone. The complication (or tumor control) probability distribution is generated using a Monte Carlo method outlined in Ref. 7. For a cohort with *npat* patients, for each DDVH file a corresponding model prediction of the NTCP is calculated, ${\text{NTCP}}_{i}$ (i=1 to *npat*), and a random number between zero and one is generated, ${\text{RN}}_{i}$. The random numbers are used to represent pseudodata of a clinical trial with *npat* patients. A complication for patient *i* is assigned if ${\text{RN}}_{i}<{\text{NTCP}}_{i}$, and thus the number of complications in this trial is equal to the number of times this inequality is true for the *npat* random numbers. This procedure is then repeated a large number of times (10 000 trials) to generate a probability distribution for the number of complications. This probability distribution provides another useful means of retrospectively comparing model predictions to actual treatment outcomes. The described Monte Carlo method of calculating this distribution is a much faster surrogate for explicit calculation of the corresponding multivariate binomial probability distribution.

#### C.5 Display and output

The main output of the TCP_NTCP_CALC is a figure containing the following items:

1. a plot of the cumulative DVH; in “group‐mode” analysis, the mean cumulative DVH is displayed;
2. text describing the location of the DDVH file (or directory) being analyzed;
3. a table that includes calculated NTCP/TCP predictions for each of the models for which parameters were available/specified; descriptors of the parameter database and the clinical data relevant to each model prediction; database descriptors of the tissue/tumor for each prediction.

**Figure 1:**
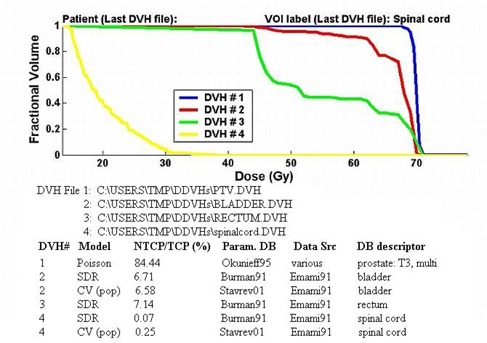
Example output from the program for a case where the user has chosen to display and analyze DDVHs for the bladder (DVH #2), rectum (DVH #3), spinal cord (DVH #4), and prostate tumor volumes (DVH #1).

More than one DVH and corresponding set of model predictions can be displayed in one figure, if desired. Figure 1 shows the program output for a case where the user has chosen to display DVHs for a prostate target, and the normal tissues of the bladder, rectum, and spinal cord. The output figure conveniently summarizes the analysis and is suitable for printing. This may be useful for archiving or consultation purposes. Analysis results are also output to a text file.

When using “group‐mode” analysis, the TCP_NTCP_CALC will also display an additional figure showing the predicted probability distribution for the number of complications in the cohort of patients described by the supplied set of DDVHs. This probability distribution is calculated and can be displayed for each of the models/parameter sets evaluated.

## III. RESULTS AND DISCUSSION

A few brief examples of potential uses of the TCP_NTCP_CALC module are demonstrated in this section.

### A.1 Retrospective analysis of treatments for a cohort of patients

**Figure 2:**
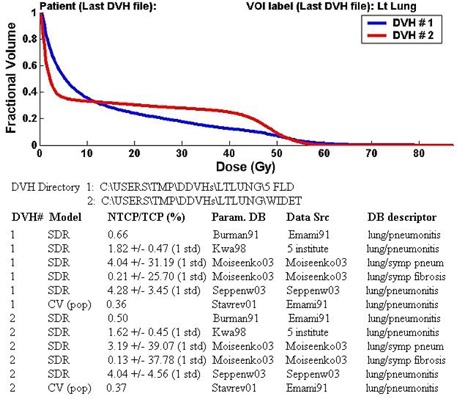
Program output after analysis of lung DVHs generated from the retrospective treatment planning of a cohort of 16 breast cancer patients using two different treatment techniques. DVH #1 (blue line) is the cumulative DVH (averaged over the 16 patients) for a “5‐field” technique, while DVH #2 (red line) is the corresponding DVH for a “wide‐tangent” technique. For each set of DVHs, radiobiological model predictions of the mean NTCP are displayed for a number of different parameter sets available in the literature.

**Figure 3:**
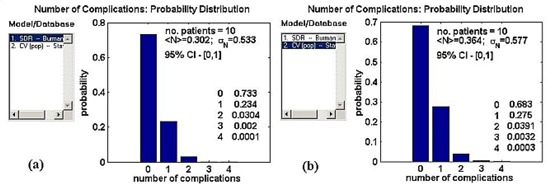
Program output displaying the distribution of the number of complications predicted from the DVHs for the mandible for a group of 10 patients based on two sets of radiobiological predictions based on the Emami et al.^(16)^ data. (a) SDR model, Burman et al.^(17)^ parameters; (b) “population” CV model, Stavrev et al.^(7)^ parameters.

The analysis of a group of DVHs corresponding to a cohort of patients treated with a given treatment technique is one useful application of the module. The program output for such an application is illustrated in Figs. 2 and 3. Figure 2 shows the results of a comparison of the lung toxicity arising from two different breast‐cancer treatment techniques: “five‐field” and “wide‐tangent.”^(32)^ The same set of 16 patients was retrospectively planned using both techniques. In Fig. 2, the mean cumulative DVH for each technique is shown and indicates that, for this example, a larger fraction of the lung is exposed to both very small doses and to large doses with the “wide‐tangent technique” (DVH #2); for example, approximately 25% less lung is exposed to doses exceeding 5 Gy, but about 8% more lung is exposed to doses exceeding 40 Gy with the “wide‐tangent” technique. For each set of DVHs, Fig. 2 also displays the mean NTCP model predictions based on SDR parameter sets from four different sources^(^ ^17^ ^,^ ^22^ ^–^ ^24^ ^)^ and one CV parameter set.^(7)^ For the shown parameter sets, the estimated mean probability of lung pneumonitis ranges from 0.4% to 4.3% for the “5‐field” technique; a similar probability of pneumonitis is predicted with the “wide‐tangent” technique, with corresponding mean NTCPs ranging from 0.4% to 4.0%. For both techniques, the NTCP predictions based on the Emami et al. (SDR and CV models) are appreciably lower than the complication probabilities estimated using more recently published SDR parameter sets.

As shown in Fig. 2, there are considerable uncertainties in the NTCP predictions based on the Moiseenko et al. and (to a lesser extent) the Seppenwoolde et al. SDR model parameters. These parameters were derived from analyses of clinical data sets consisting of 55 and 382 patients, respectively. This once again underlines the challenge in generating precise radiobiological predictions: The statistics and diversity of clinical data are in general insufficient to define narrow confidence intervals for parameter estimates. The uncertainties in the NTCP predictions based on the Kwa et al. model parameters are significantly lower than the other error estimates. However, this is at least partly due to the fact that Kwa et al. fixed the parameter n=1, which also led to tighter confidence intervals for the other two parameters, *m* and $D_{50}$. The validity of these NTCP predictions is thus implicitly dependent on the validity of the assumption that the mean lung dose is an accurate predictor of lung response to a heterogeneous dose delivery lung. Note also that the shown Kwa et al. NTCP predictions do not include the institute‐dependent offset of 0% to 11% in the NTCP reported in their work.

Fig. 3 shows the second program output, the predicted probability distribution of the number of complications, when a group of 10 DDVHs describing dose distributions in the mandible is analyzed. For this normal tissue, the program database contains the SDR and CV parameter sets based on the Emami et al. data. As shown, the complication distributions are similar for these two parameter sets: Using the SDR parameters, the program predicts probabilities of 73%, 23%, and 3% for observing zero, one, and two or more complications; using the CV parameters, the corresponding probabilities are 68%, 28%, and 4%. The mean, standard deviation, and 95% confidence interval of these distributions are also included in the output and are as shown.

### A.2 Testing of the sensitivity of model predictions to parameter values

**Figure 4:**
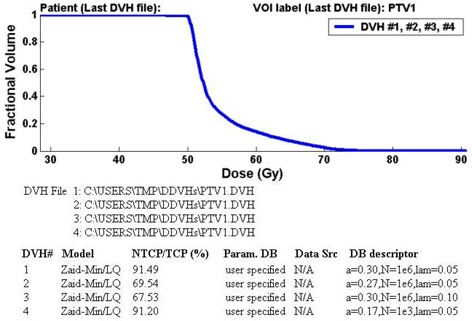
Program output displaying TCP predictions for the same DDVH (i.e., DVHs #1 to #4 are the same) for four sets of user‐specified parameters: (i) $\alpha =0.30\;{\text{Gy}}^{-1},\beta =0.03\;{\text{Gy}}^{-2},N=10^{6},\lambda =0.05\;{\text{days}}^{-1},n=25$ fractions; (ii) same as (i), but with slightly decreased cellular radiosensitivity, $\alpha =0.27\;{\text{Gy}}^{-1},\beta =0.027\;{\text{Gy}}^{-2}$; (iii) same as (i), but with the rate of repopulation doubled, $\lambda =0.10\;{\text{days}}^{-1}$; (iv) $\alpha =0.17\;{\text{Gy}}^{-1},\beta =0.017\;{\text{Gy}}^{-2},N=10^{3},\lambda =0.05\;{\text{days}}^{-1},n=25$ fractions.

The TCP_NTCP_CALC program is also useful to those seeking to test the sensitivity of model predictions to different model parameter values. Figure 4 displays the program output for the case when a user has chosen to specify four sets of parameter values for the Zaider‐Minerbo/LQ TCP model, in the analysis of a given tumor DDVH file. The first time the following parameters were specified: LQ cellular radiosensitivity values of $\alpha =0.30\;{\text{Gy}}^{-1}$ and $\beta =0.03\;{\text{Gy}}^{-2}$ (i.e., α/β=10 Gy); $N=10^{6}$ for the number of tumor clonogens; $\lambda =0.05\;{\text{days}}^{-1}$, which corresponds to a potential doubling time (ln2λ) of about 14 days; and n=25 fractions. These parameters lead to a predicted TCP of 91.5%. For the second set of parameters, the radiosensitivity is reduced by 10%, with values of $\alpha =0.27\;{\text{Gy}}^{-1}$ and $\beta =0.027\;{\text{Gy}}^{-2}$ being specified (all other parameters the same as the first set). This reduces the predicted TCP by 22% to 69.5%, demonstrating the considerable sensitivity of the TCP calculation to small changes or uncertainties in the cellular radiosensitivity. A similar reduction of 24% in the TCP (to 67.5%) is also predicted if instead of changing the radiosensitivity, the repopulation rate is doubled to $\lambda =0.10\;{\text{days}}^{-1}$ (parameter set #3). Use of the fourth set of parameter values—$\alpha =0.17\;{\text{Gy}}^{-1},\beta =0.017\;{\text{Gy}}^{-2},N=10^{3},\lambda =0.05\;{\text{days}}^{-1},n=25$ fractions—is used to describe a much smaller tumor with increased cellular radio resistance. The predicted TCPs of 91.2% and 91.5% for the fourth and first sets of parameters, respectively, are nearly the same. This indicates that the 1000‐fold decrease in the size of the tumor can be offset by a reduction in the radiosensitivity parameters of only 43%. Indirectly this also suggests, as has been observed in numerous radiobiological modeling works, that in a heterogeneous tumor, tumor response is determined mainly by the most radio‐resistant subpopulation within the tumor.

## IV. CONCLUSION

We developed an NTCP‐TCP estimation module, TCP_NTCP_CALC, which can be used as a research tool and as a clinical aid. Our module can assist in the evaluation of treatment plans by conveniently providing access to current radiobiological model predictions. It also provides a means of assessing the reliability and utility of common radiobiological models, both by facilitating comparison of model predictions (based on available clinical data) to actual clinical outcomes and by testing of the sensitivity of model predictions to uncertainties in the model parameters.

## ACKNOWLEDGMENTS

This research was supported by studentships from the Alberta Heritage Foundation for Medical Research and the Alberta Cancer Board (BW), and the Alberta Cancer Board RIP grant RI‐218.
